# Supplementary material for: The Periodontopathic Pathogen, Porphyromonas gingivalis, Involves a Gut Inflammatory Response and Exacerbates Inflammatory Bowel Disease
Source: Pathogens. 2022 Jan 11;11(1):84. doi: 10.3390/pathogens11010084 (PMC8779656; doi:10.3390/pathogens11010084)
Supplement: Supplementary file 1 [file pathogens-11-00084-s001.zip › pathogens-1521717-supplementary.pdf]

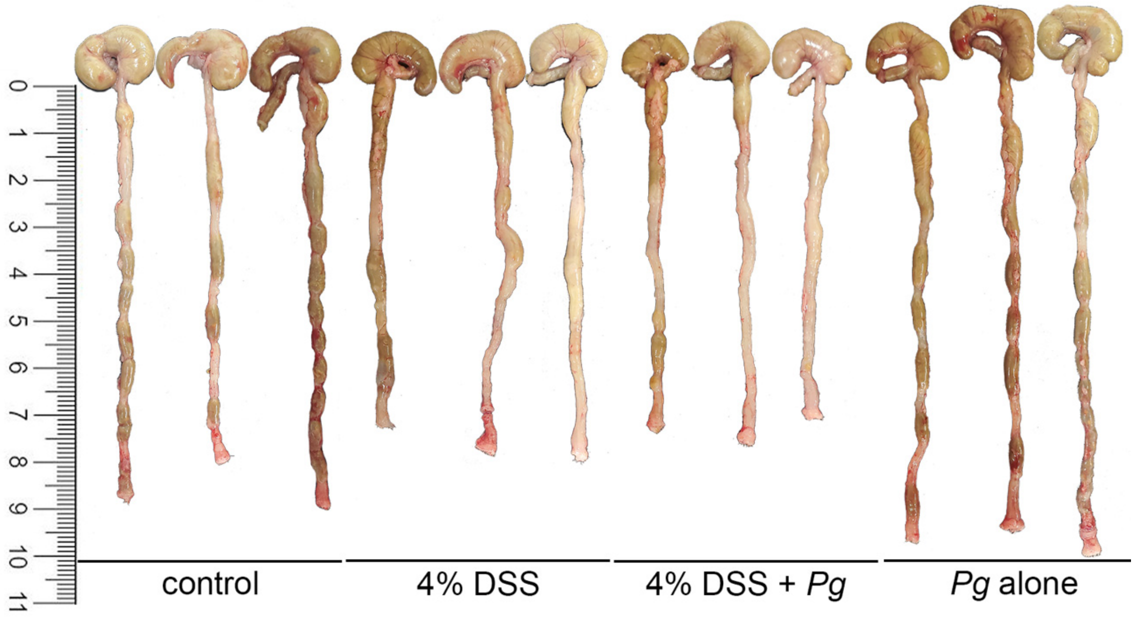

Figure S1. The picture of all the colons in each group. (**A**) Dissected colons of control group. The lengths were 9.0 cm, 8.34 cm, and 9.04 cm, respectively. (**B**) Dissected colons of 4% DSS treated group. The lengths were 7.58 cm, 8.58 cm, and 7.92 cm, respectively. (**C**) Dissected colons of 4% DSS plus *Pg* group. The lengths were 7.06 cm, 7.65 cm, and 7.18 cm, respectively. (**D**) Dissected colons of *Pg* alone group. The lengths were 9.77 cm, 9.27 cm, and 10.09 cm, respectively. DSS, dextran sulfate sodium; *Pg*, *Porphyromonas gingivalis*.
